# Invasive non-typeable *Haemophilus influenzae* infection due to endometritis associated with adenomyosis

**DOI:** 10.1186/s12879-020-05193-2

**Published:** 2020-07-16

**Authors:** Yoshito Nishimura, Hideharu Hagiya, Kaoru Kawano, Yuya Yokota, Kosuke Oka, Koji Iio, Kou Hasegawa, Mikako Obika, Tomoko Haruma, Sawako Ono, Hisashi Masuyama, Fumio Otsuka

**Affiliations:** 1 Department of General Medicine, Okayama University Graduate School of Medicine, Dentistry and Pharmaceutical Sciences, 2-5-1 Shikata-cho, Kita-ku, Okayama, 700-8558 Japan; 2 Microbiology Division, Clinical Laboratory, Okayama University Hospital, Okayama, Japan; 3 Department of Obstetrics and Gynecology, Okayama University Graduate School of Medicine, Dentistry and Pharmaceutical Sciences, Okayama, Japan; 4 Department of Pathology, Okayama University Graduate School of Medicine, Dentistry and Pharmaceutical Sciences, Okayama, Japan

**Keywords:** Non-typable *Haemophilus influenzae*, Bacteremia, β-Lactamase-nonproducing ampicillin-resistance, Adenomyosis, Case report

## Abstract

**Background:**

The widespread administration of the *Haemophilus influenzae* type b vaccine has led to the predominance of non-typable *H. influenzae* (NTHi). However, the occurrence of invasive NTHi infection based on gynecologic diseases is still rare.

**Case presentation:**

A 51-year-old Japanese woman with a history of adenomyoma presented with fever. Blood cultures and a vaginal discharge culture were positive with NTHi. With the high uptake in the uterus with ^67^Ga scintigraphy, she was diagnosed with invasive NTHi infection. In addition to antibiotic administrations, a total hysterectomy was performed. The pathological analysis found microabscess formations in adenomyosis.

**Conclusions:**

Although NTHi bacteremia consequent to a microabscess in adenomyosis is rare, this case emphasizes the need to consider the uterus as a potential source of infection in patients with underlying gynecological diseases, including an invasive NTHi infection with no known primary focus.

## Background

*Haemophilus influenzae*, a gram-negative coccobacillus, is a common cause of respiratory tract infections (e.g., pneumonia) and meningitis, particularly in children [1–3]. Although *H. influenzae* type b (Hib) is a notoriously virulent serotype of this species [4], the introduction of routine conjugate Hib vaccination led to a decrease in the number of cases of Hib infection [5]. Over the past decades, however, this decrease was paralleled by an increase in the frequency of non-typable *H. influenzae* (NTHi) infection, which now accounts for the majority of invasive *H. influenzae* infection cases [6–9].

In Japan, a recent nationwide population-based surveillance study revealed that NTHi and *H. influenzae* type f became the predominant isolates associated with invasive *H. influenzae* infection after the introduction of the Hib vaccine [10]. Previous studies have identified invasive NTHi infection as a cause of sepsis in adults, including pregnant women [11]. While previous reports have established the urogenital tract as a potential cause of invasive *H. influenzae* infection, no reports have described a specific association of NTHi infection with adenomyoma. Here, we report a case of invasive NTHi infection associated with a massive adenomyosis in an immunocompetent Japanese woman.

## Case presentation

A 51-year-old Japanese woman with a history of adenomyoma was admitted to our hospital with fever. Four months before the admission, she had been referred to a gynecologist because of menorrhagia and metrorrhagia and was clinically diagnosed with a massive polypoid adenomyoma (PAM). Three days prior to hospital admission, she complained of fever as high as 40 °C, chills, and lower abdominal discomfort and revisited a nearby clinic, which referred her to our hospital upon suspicion of uterine infection.

During the examination, the patient was alert and oriented. She had a body temperature of 39.4 °C and a respiratory rate of 26/min. A physical examination revealed mild lower abdominal tenderness upon palpation. Other physical examinations, including a pelvic examination by a gynecologist, were noncontributory. Her medical history was significant for a massive adenomyoma but otherwise unremarkable. Blood testing upon admission revealed an elevated white blood cell count of 1.61 × 10^4^/μL with neutrophil predominance and an elevated C-reactive protein concentration of 21.13 mg/dL. The serum immunoglobulin and complement component concentrations were within normal ranges. A urinalysis revealed no pyuria or bacteriuria, and direct urine polymerase chain reaction analyses for chlamydia and gonorrhea were negative. A non-contrast computed tomography (CT) scan confirmed the massive adenomyosis of the uterus but revealed no evidence of uterine abscess formation, pyelonephritis, other sources of fever, or asplenia. Intravenous ceftriaxone treatment was initiated after two sets of blood cultures, a urine culture, and a vaginal discharge culture were obtained.

One day after admission, the blood culture analysis yielded a positive result for Gram-negative coccobacilli (BD BACTEC™ FX blood culture system, Becton Dickinson, Sparks, MD, USA). The organism was subsequently identified as *H. influenzae* according to a positive X and V factor test (*Haemophilus* Differentiating Medium, Kyokuto Pharmaceutical Industrial Co., Ltd., Tokyo, Japan)*,* biochemical assay (ID test HN-20 rapid, Nissui Pharmaceutical, Tokyo, Japan), and mass spectrometry analysis (MALDI Biotyper, Bruker Daltonics Inc., Billerica, MA, USA), which led to the diagnosis of invasive *H. influenzae* infection.

On the second day after admission, the vaginal discharge bacterial culture also yielded a positive result indicating *H. influenzae.* Both the blood and vaginal specimens were subjected to an antimicrobial resistance pattern analysis (Dryplate broth microdilution panels, Eiken Chemical Co., Ltd., Tokyo, Japan) and slide agglutination test with six specific antisera against each type of bacterial capsular polysaccharide (Bacterial antisera “SEIKEN”, Denka Seiken Co., Ltd., Japan), which identified the causative organism as NTHi with a β-lactamase-nonproducing ampicillin-resistance pattern. The result of antimicrobial susceptibility testing is shown in Table 1. The antimicrobial susceptibility profiles were identical for both isolates from the blood culture and vaginal discharge.

**Table 1:** Minimal inhibitory concentrations (MICs) of antibiotics for *Haemophilus influenzae* detected in the blood culture

Antibiotics	MICs^a^ (μg/mL)	Interpretation^b^
Ampicillin	4	R
Ampicillin/Sulbactam	4	R
Cefotaxime	1	S
Cefepime	2	S
Imipenem	1	S
Meropenem	≤0.25	S
Levofloxacin	≤0.25	S

^a^MICs were determined by broth microdilution for the clinical isolate

^b^Interpretation according to CLSI Performance Standards for Antimicrobial Susceptibility Testing (M100-S29). CLSI supplement M100. Wayne, PA: Clinical and Laboratory Standards Institute; 2020

**Fig. 1:**
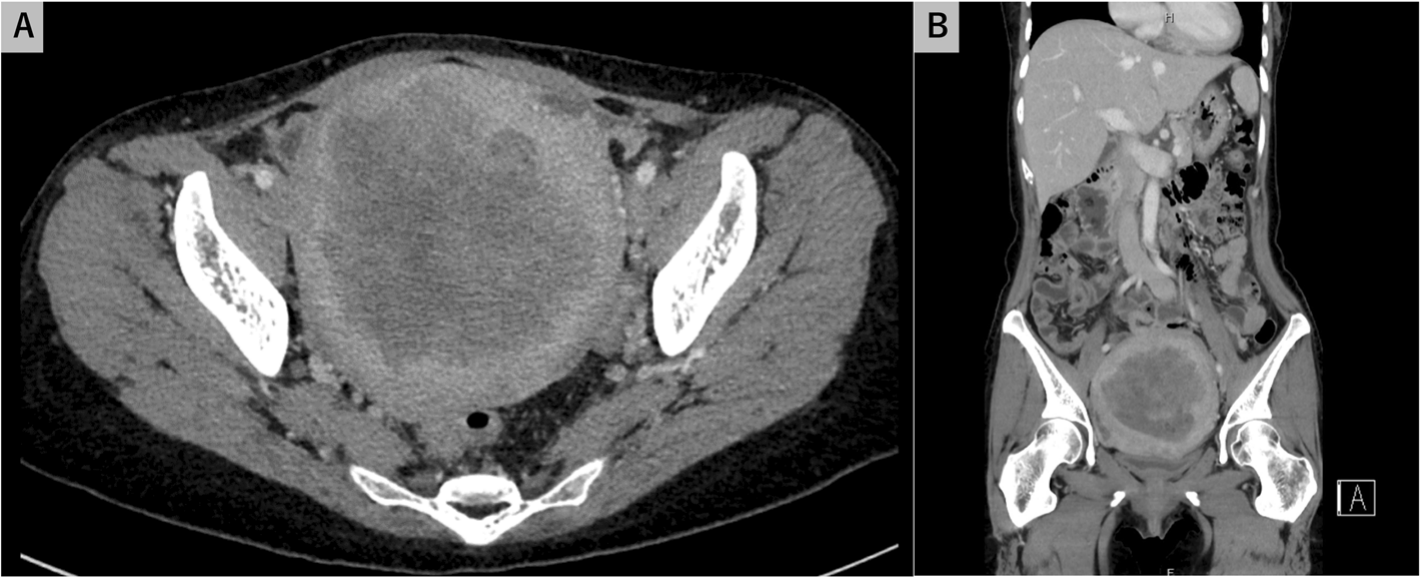
Contrast-enhanced computed tomography (CT) of the uterus after hospital admission. Although uterine enlargement was visible, no abscess formation was detected. **a** horizontal view; **b** coronal view

**Fig. 2:**
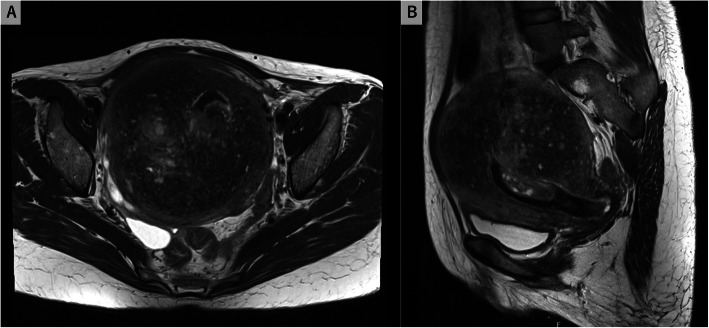
Contrast-enhanced magnetic resonance imaging (MRI) of the pelvis. Despite a high clinical suspicion of *H. influenzae* infection in the uterus, T2-weighted MRI revealed no evidence of uterine abscess formation. The uterus was enlarged due to a known adenomyoma. **a** horizontal view; **b** sagittal view

**Fig. 3:**
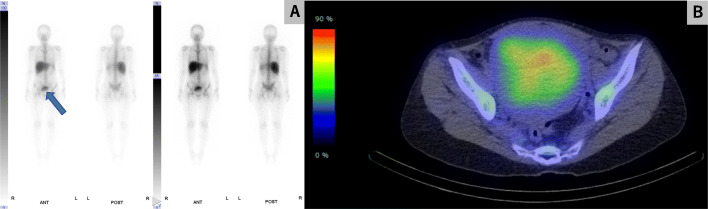
Gallium-67 (^67^Ga) scintigraphy of the uterus after prednisone treatment. ^67^Ga scintigraphy revealed high uptake in the intrauterine cavity (arrow), which suggested an active infection in the uterus. High ^67^Ga uptake was not observed in other organs. **a** anterior and posterior images of ^67^Ga scintigraphy; **b** horizontal view

**Fig. 4:**
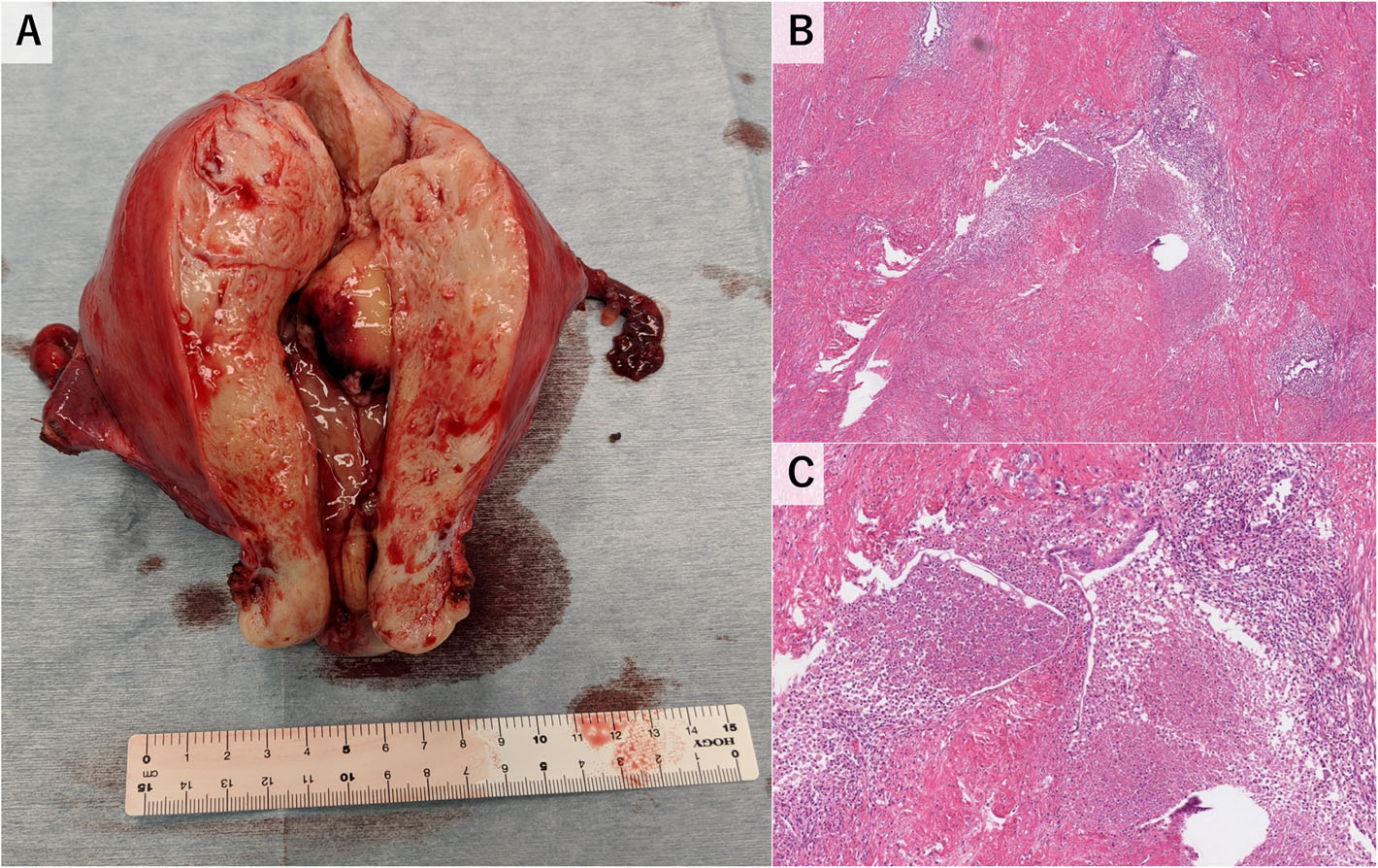
Macro- and micropathological findings of the uterus. **a** Gross findings of the excised uterine specimen. The uterus was enlarged, and a polyp protruding into the endometrial cavity. **b** Microabscess formations and necrosis were noted next to adenomyosis. Magnification × 100. **c** Magnification × 400

The patient remained febrile for 3 consecutive days, despite continued intravenous ceftriaxone therapy. A pelvic CT scan and magnetic resonance imaging with contrasts were performed to detect abscess formation in the uterus. However, the results were inconclusive (Figs. 1 and 2). Intravenous ceftriaxone therapy was continued based on the drug susceptibility test results. The patient became afebrile 7 days after admission. Meanwhile, we performed gallium-67 (^67^Ga) scintigraphy to evaluate the uterine infection, as no other possible origin of the febrile infection had been identified. The analysis revealed high ^67^Ga uptake confined to the internal layer of the uterus (Fig. 3). A total hysterectomy was performed 17 days after admission due to the high likelihood of uterine *H. influenza* infection. Although no grossly apparent uterine sources of infection were identified, the pathological analysis revealed microabscess formation in the enlarged uterus (Fig. 4), which was compatible with a uterine *H. influenzae* infection based on the underlying adenomyosis. The pathological diagnosis also revealed an early-stage endometrioid carcinoma. The patient’s postoperative course was uncomplicated, and she did not receive additional antibiotic administration. She was discharged home a month after admission and was instructed to participate in outpatient gynecological follow-up.

## Discussion and conclusions

Although *H. influenzae* typically colonizes the respiratory tracts, it has been reported that a few women might carry NTHi in their genital tracts [12]. The comparatively widespread nature of NTHi consequent to the use of Hib vaccine in recent years emphasizes the need to familiarize ourselves with this important pathogen [7, 8]. Our present case not only involved a rare occurrence of an invasive *H. influenzae* infection associated with adenomyosis, but also presents several lessons for those dealing with infectious disease in their daily practices. First, the presence of a gynecologic disease, such as adenomyosis, may become a nidus of complicated NTHi infection. Second, even in a case involving a complicated uterine infection, appropriate cultures and a pathological diagnosis are essential tools for detecting the sources of infections.

Previously, only a few articles reported association of gynecological diseases with *H. influenzae* infection. For example, Chen et al. reported a case of *H. influenzae* vulvovaginitis in a prepubertal girl [13]. Martin and colleagues described a case of NTHi bacteremia due to acute endometritis, in which the uterus appeared to be the nidus of NTHi infection with no known primary focus [14]. Also, it has been reported that *Haemophilus quentini*, a NTHi biotype IV isolate, may have a predilection for female urogenital tract [15]. While identification of *H. quentini* isolates are not currently feasible at frontline laboratories, it is worth noting that NTHi could be an important causative pathogen of gynecological infections. Adenomyosis is a common gynecological disease involving ectopic endometrial glands and stroma within the myometrium. A PAM is defined as an adenomyoma that has protruded into the endometrial cavity [16], and can be divided histopathologically into typical and atypical types. An atypical PAM comprises a mixture of epithelial and mesenchymal components, and different degrees of atypia, including endometrioid carcinoma, may be present within the endometrial glands [17]. The diagnosis of PAM is difficult, and pathological analysis is imperative. Although this case was finally diagnosed as adenomyosis, based on previous reports that have described invasive NTHi cases attributed to endometriosis and pregnancy [6, 14], we may infer that any anatomical abnormality in the female reproductive organs could be a nidus of bacterial infection.

The determination and diagnosis of the uterus as a source of infection, as in this case, remains challenging, despite the importance of this organ with regard to invasive NTHi infection. As per the World Health Organization (WHO), both the collection of a swab bacterial culture and a pelvic examination are essential when evaluating a case with a high clinical suspicion of a reproductive system infection [18]. Moreover, the combination of pathological analyses is encouraged to strengthen the diagnostic accuracy, as underscored by a previous report in which *H. influenzae* was detected in an endometrial tissue biopsy specimen [14]. In our case, the antimicrobial resistance pattern and phenotypes of *H. influenzae* detected in the blood cultures coincided with those in the vaginal discharge culture. The addition of ^67^Ga scintigraphy and pathological analyses of the uterus helped to confirm the diagnosis.

In conclusion, we have presented a case of invasive NTHi infection associated with adenomyosis. Clinicians faced with a patient with no known primary focus of *H. influenzae* bacteremia and an underlying gynecological disease should consider the uterus as a possible source of infection.

## Data Availability

Not applicable.

## Abbreviations

^67^Ga: Gallium-67
CT: Computed tomography
Hib: *H. influenzae* type b
NTHi: Non-typable *Haemophilus influenzae*
PAM: Polypoid adenomyoma
WHO: World Health Organization
